# Curcumin: Could This Compound Be Useful in Pregnancy and Pregnancy-Related Complications?

**DOI:** 10.3390/nu12103179

**Published:** 2020-10-17

**Authors:** Tiziana Filardi, Rosaria Varì, Elisabetta Ferretti, Alessandra Zicari, Susanna Morano, Carmela Santangelo

**Affiliations:** 1 Department of Experimental Medicine, Section of Medical Pathophysiology, Food Science and Endocrinology, Sapienza University, Viale Regina Elena 324, 00161 Rome, Italy; susanna.morano@uniroma1.it; 2 Center for Gender-Specific Medicine, Gender Specific Prevention and Health Unit, Istituto Superiore di Sanità, Viale Regina Elena 299, 00161 Rome, Italy; rosaria.vari@iss.it (R.V.); carmela.santangelo@iss.it (C.S.); 3 Oncogenomic Unit, Department of Experimental Medicine, Sapienza University, Viale Regina Elena 324, 00161 Rome, Italy; elisabetta.ferretti@uniroma1.it; 4 Department of Experimental Medicine, 2nd Section of Cell Pathology, Sapienza University, Viale Regina Elena 324, 00161 Rome, Italy; alessandra.zicari@uniroma1.it

**Keywords:** curcumin, pregnancy, pregnancy complications, postpartum depression, fetal development, preterm birth, adverse effects

## Abstract

Curcumin, the main polyphenol contained in turmeric root (*Curcuma longa*), has played a significant role in medicine for centuries. The growing interest in plant-derived substances has led to increased consumption of them also in pregnancy. The pleiotropic and multi-targeting actions of curcumin have made it very attractive as a health-promoting compound. In spite of the beneficial effects observed in various chronic diseases in humans, limited and fragmentary information is currently available about curcumin’s effects on pregnancy and pregnancy-related complications. It is known that immune-metabolic alterations occurring during pregnancy have consequences on both maternal and fetal tissues, leading to short- and long-term complications. The reported anti-inflammatory, antioxidant, antitoxicant, neuroprotective, immunomodulatory, antiapoptotic, antiangiogenic, anti-hypertensive, and antidiabetic properties of curcumin appear to be encouraging, not only for the management of pregnancy-related disorders, including gestational diabetes mellitus (GDM), preeclampsia (PE), depression, preterm birth, and fetal growth disorders but also to contrast damage induced by natural and chemical toxic agents. The current review summarizes the latest data, mostly obtained from animal models and in vitro studies, on the impact of curcumin on the molecular mechanisms involved in pregnancy pathophysiology, with the aim to shed light on the possible beneficial and/or adverse effects of curcumin on pregnancy outcomes.

## 1. Introduction

Maternal nutrition is an essential and modifiable environmental factor that deeply influences maternal and offspring health in the short and long-term [1,2,3,4,5,6]. Genetics, nutrition, and other environmental factors significantly contribute to the physiological immune and metabolic modifications occurring in pregnancy, to favor maternal adaptation to the growing and developing fetus. Maternal malnutrition adversely affects these dynamic processes by acting on the mechanisms related to the nutritional programming, including nutrition sensing signals, epigenetic regulation, gut microbiome, as well as on the nutrient-nutrient and nutrient-drug interactions, modulating maternal and fetal genes in a sex-specific manner [3,6,7,8,9].

Over the last decades, the advantages of a healthy diet, rich in fruit and vegetables, have been widely explored, highlighting that culinary herbs and spices might also effectively reduce the risk of developing chronic diseases [10]. Among them, curcumin, a compound extracted from the rhizome of *Curcuma longa*, has been extensively studied in light of a wide range of properties, including anti-inflammatory, antioxidant, anti-toxicant, antiapoptotic, immunomodulatory, neuroprotective, hepatoprotective, antiangiogenic, anti-hypertensive, and antidiabetic activities, emerging as a candidate therapeutic agent for several diseases [10,11,12,13]. Data from animal and in vitro studies provided evidence that curcumin might be effective in counteracting the adverse programming processes in pregnancy.

The known pathophysiological mechanisms underlying pregnancy and the most common pregnancy-related complications, such as gestational diabetes mellitus (GDM) [14], hypertension and preeclampsia [15], fetal growth disorders [16], as well as the damage induced by natural and chemical toxic agents [12] seem to be positively modulated by curcumin, although observed in in vitro and animal studies.

Additionally, promising results from preclinical studies on the use of curcumin in different neurological disorders [17] suggest a potential role in the treatment of post-partum depression (PPD) as well, a largely underestimated pregnancy-related disorder [18].

Harmful effects of curcumin on embryo development in the early stages of pregnancy have also been observed in animal studies [19]. Hence, the increasing consumption of natural products during pregnancy requires particular attention, considering the complexity of the largely unknown processes underlying maternal adaptation and fetal development.

We conducted a comprehensive literature search until 22 July 2020 using PubMed; and found a good number of articles in English, using the keywords “pregnancy”, “pregnancy complications”, “gestational diabetes”, “preeclampsia”, “reproductive toxicity”, “post-partum depression”, “placenta”, “oocyte”, “blastocyst”, “embryo”, “preterm labor”, “fetal growth and development”, in combination with the keywords “curcumin” and “dietary curcumin”. The aim of this review is to provide an overview of both the potential health benefits and the possible adverse effects of curcumin in pregnancy and pregnancy-related complications.

## 2. Curcumin: Functions, Bioavailability, and Delivery

Curcumin, also called diferuloylmethane, is a lipophilic polyphenol extracted from the rhizome of *Curcuma Longa* (commonly known as turmeric). It has been widely used in traditional Indian and Chinese medicine for thousands of years [20]. The pharmacological effects of turmeric have been attributed mainly to curcuminoids, comprising curcumin and two related compounds, demethoxycurcumin and bisdemethoxycurcumin, which are contained in commercial curcumin [21]. Curcumin is a potent anti-inflammatory and antioxidant agent that exerts a myriad of biological activities by influencing multiple signaling pathways [10,11,13,22]. Curcumin is able to interact with a large number of molecular and cellular targets (as summarized in this recent review [13]) and regulates gene expression also by modulating epigenetic modifications (i.e., DNA methylation, histone modification, and microRNA expression) [23,24]. This compound, by mutually interacting with intestinal microflora, ameliorates gut microbiome dysbiosis, and influences the “gut–brain–microflora axis” to preserve and favor brain health [25,26]. The overall result of these different activities is the improvement in several disease states, including inflammatory, metabolic, endocrine, cardiovascular, gastrointestinal, neurological, respiratory, viral, skin diseases, and cancer, as highlighted by the impressive number of in vitro and in vivo studies summarized in recent papers [13,24,27,28]. Numerous clinical trials have shown good tolerability, safety, and efficacy of curcumin in the treatment of multiple chronic diseases—including cardiovascular diseases, diabetes, neurodegeneration, arthritis, and cancer—at doses up to 6–12 g/day [10,11,13]. In light of this, the United States Food and Drug Administration (FDA) has “Generally Recognized As Safe” (GRAS) curcumin as an ingredient in various food categories (0.5–100 mg/100 g) [29]; and the European Food Safety Authority (EFSA) Panel on Food Additives and Nutrient Sources added to Food (ANS), defined the Allowable Daily Intake (ADI) value of 0–3 mg/kg bw/day of curcumin as a food additive [30]. However, despite its potential therapeutic benefits, curcumin is poorly bioavailable due to its rapid metabolism, and the small portion of substance that is absorbed is extensively bio-transformed into its water-soluble metabolites, glucuronides, and sulfates [10]. Therefore, several strategies have been developed to enhance its bioavailability and efficacy, to increase oral and gastro-intestinal absorption, and to reduce the clearance from the body [31,32,33]. For this purpose, taking into consideration that curcumin is fat-soluble, several delivery systems have been developed to obtain a number of formulations by mixing curcumin with different materials, including adjuvants, such as piperine [32,33]. Micelles, liposomes, phospholipid complexes, phytosomes, emulsions, microemulsions, nano-emulsions, solid lipid nanoparticles, nanostructured lipid carriers, biopolymer nanoparticles, and microgels represent different and recent technical approaches to encapsulate curcumin [32,33,34], although further studies are needed to evaluate their effectiveness and safety as potential health-promoting compounds in humans.

## 3. Role of Curcumin in Pregnancy

### 3.1. Altered Glucose Metabolism

It is well known that dynamic changes in insulin sensitivity take place during healthy pregnancy to allow adequate supply to the growing fetus [35]. In pregnancy, several players, including hormones, cytokines, and metabolic factors, contribute to the development of insulin resistance through complex mechanisms, not yet completely understood [36,37]. Maternal obesity, related to unhealthy diet and lifestyle, can negatively affect insulin sensitivity leading to the development of GDM and type 2 diabetes (T2D), with serious short and long-term health consequences for both the mother and the offspring [38,39]. Recent evidence emphasized the anti-hyperglycemic activity of curcumin, both in animals and humans [40]. Specifically, this compound had the capability to improve glucose uptake, insulin sensitivity, and pancreatic β-cell function, as well as liver and kidney function, and to reduce glucose and lipid levels, oxidative stress, and inflammation [41], by interacting with almost all the players involved in these processes, as demonstrated in in vitro studies [13,41].

As regards human studies, the effects of curcumin supplementation have been evaluated in several randomized controlled trials. A recent intervention study showed that 1500 mg/day curcumin supplementation (500 mg capsules: 347 mg of curcumin, 84 mg of demethoxycurcumin, and 9 mg of bisdemethoxycurcumin) for 10 weeks reduced triglycerides (TG) and C-reactive protein (CRP), and increased adiponectin levels [42], whereas 500 mg/day curcumin co-administered with piperine 5 mg/day for three months was able to reduce blood glucose, C-peptide, glycated hemoglobin (HbA1c), alanine aminotransferase (ALT) and aspartate aminotransferase (AST), in patients with T2D [43]. Another study showed that the daily ingestion of 2100 mg turmeric powder for eight weeks resulted in a reduction in body weight, low density lipoprotein-cholesterol (LDL-c), and TG levels, with no significant effects on glycemia, CRP, and HbA1c, in hyperlipidemic T2D patients [44]. In obese women with polycystic ovary syndrome (PCOS), 1000 mg/day curcumin supplementation (500 mg twice daily: 70–80% curcumin, 15–20% demethoxycurcumin and 2.5–6.5% bisdemethoxycurcumin) for six weeks improved serum insulin and the Quantitative Insulin Sensitivity Check Index [45]. A recent meta-analysis reported that curcumin intake was associated with reduced body mass index (BMI), body weight, body fat, leptin value, and increased adiponectin levels in patients with metabolic syndrome and related disorders [46]. Overall, the dosage and duration of curcumin supplementation appear to differently modulate glucose metabolism in humans.

A recent promising approach to treat hyperglycemia consists of combining the effects of curcumin and the ongoing antidiabetic agents, as observed in diabetic rats treated with a combination of curcumin and metformin. Specifically, this association improved hyperglycemia, dyslipidemia, and oxidative stress, increasing the activity of the antioxidant enzyme paraoxonase 1 (PON1), in diabetic rats [47].

Dietary bioactive compounds might have beneficial effects on GDM [5,48]. In particular, curcumin appeared to improve GDM and GDM-related complications in a recent study in a mouse model. Specifically, C57 BL/KsJdb/+ diabetic pregnant mice were supplemented with different curcumin dosages: 50 mg/kg and 100 mg/kg/day, from gestational day zero (GD 0) to GD20. Results showed that 100 mg/kg curcumin significantly reduced blood glucose and insulin levels, increased hepatic glycogen content, and improved oxidative stress by reducing thiobarbituric acid reactive substance (TBARS) and increasing glutathione (GSH) levels, superoxide dismutase (SOD), and catalase (CAT) activities in the liver of diabetic pregnant mice at gestational day 20. The reduced 5’ adenosine monophosphate-activated protein kinase (AMPK) and increased Histone Deacetylase 4 HDAC4 activities observed in GDM liver were reverted by curcumin treatment. Furthermore, curcumin positively influenced the offspring of mothers with GDM, restoring litter size and birth weight, and inducing the reduction of glucose-6-phosphatase (G6Pase) expression and activity in the liver [14] (Table 1). Congenital birth defects, including neural tube defects (NTD), occur more often in the offspring of diabetic mothers. In a recent study, mouse embryos (at E8.5 of development) were cultured for 24 h with 100 mg/dL glucose, in the absence or presence of curcumin (10 and 20 μM). Remarkably, 20 μM curcumin was able to reduce the rate of embryos with NTD induced by high glucose. Curcumin reduced high glucose-induced oxidative and nitrosative stress [i.e., decreased 4-hydroxynonenal (4-HNE), nitrotyrosine levels, and lipid hydroperoxide (LPO)], as well as endoplasmic reticulum (ER) stress (i.e., decreased expression of ER-markers stress such as phosphorylated protein kinase-like endoplasmic reticulum kinase (p-PERK), phosphorylated inositol-requiring protein-1α (p-IRE1α), phosphorylated eukaryotic initiation factor 2α (p-eIF2α), C/EBP-homologous protein (CHOP), binding immunoglobulin protein (BiP), and x-box binding protein 1 (XBP1). Moreover, 20 μM curcumin inhibited the cleavage of pro-apoptotic caspases (i.e., casp-3 and -8) [49]. Although the results from preclinical studies are overall promising, further research is needed to better understand the molecular mechanisms underlying diabetic complications, as well as the pharmacodynamics and pharmacokinetics of curcumin in pregnancy, to conceivably employ this compound as a therapeutic agent for human pregnancy complications.

### 3.2. Cardiovascular Disorders

Critical changes in the cardiovascular system occur in physiological pregnancy, to ensure maternal and fetal adaptation to the increased metabolic demand and to guarantee adequate uteroplacental circulation for fetal growth. A healthy pregnancy is hallmarked by systemic vasodilatation, significantly related to the high levels of estrogen and progesterone. Cardiac output and heart rate rise during gestation and the activation of the renin-angiotensin-aldosterone system leads to a significant increase in total blood volume. Alterations in these processes are associated with maternal and fetal morbidity and mortality [69]. Obesity, older maternal age, and diabetes mellitus increase the risk of cardiovascular diseases in pregnancy (1–4%), with a higher prevalence when including hypertensive disorders—chronic hypertension, pregnancy-induced hypertension, pre-eclampsia, and HELLP syndrome (hemolysis, elevated liver enzymes, and low platelet count) [70]. Considering the anti-inflammatory, antioxidant, and antiangiogenic activities observed in several studies, curcumin is a potential therapeutic compound in cardiovascular disorders [71].

Preeclampsia (PE) is a systemic syndrome characterized by hypertension and proteinuria, which begins after 20 weeks of gestation; it occurs in 2–8% of pregnancies, and it is a leading cause of maternal and fetal morbidity and mortality [72]. Although the pathophysiology of PE remains to be elucidated, alterations in maternal vascular physiology have been described, leading to a generalized vasoconstrictive state, systemic oxidative stress, inflammation, and endothelial cell dysfunction, with severe adverse effects on the placenta, one of the major organs that develops after conception [73,74]. Strategies to reverse or arrest the pathological processes of PE are aimed at reducing excessive inflammatory response, micro-emboli formation, and vasoconstriction by using specific drugs or natural products [75]. For this purpose, studies in animal models have been performed. It has been observed that in lipopolysaccharides (LPS)-treated pregnant rats to create a PE model (LPS 0.5 μg/kg on gestational day 5), the administration of curcumin (0.36 mg/kg, from GD 0 to GD18) improved hypertension, proteinuria, and renal damage, and reduced serum levels of IL-6 and monocyte chemoattractant protein-1 (MCP-1). Curcumin treatment ameliorated inadequate trophoblast invasion and spiral artery remodeling, significant histopathological alterations observed in PE. Analysis of placental tissue showed that curcumin administration decreased the LPS-induced expression of the inflammatory molecules toll-like receptor (TLR)-4, IL-6, and the proinflammatory transcription factor NF-kB. According to the obtained data, the authors hypothesized that curcumin may positively modulate the cascade of different signaling pathways involved in PE development [50]. Similar results were obtained in a mouse model of LPS-induced PE. In this study, in addition to blood pressure and proteinuria reduction, curcumin increased the number of live pups, fetal and placental weight, and decreased fetal desorption. These effects were associated with the inhibition of placental expression of TNF-α, IL-1β, IL-6 cytokines, and MCP-1 and MIP-1 chemokines, and with a reduction in macrophage infiltration. The reduced inflammatory status was accompanied by increased activation of the serine/threonine-specific protein kinase Akt, involved in cellular proliferation [51]. Neo-vascularization is a critical event mediated by several angiogenic factors—including the vascular endothelial growth factor (VEGF), fibroblast growth factors (FGFs), matrix metalloproteinases (MMPs)—and inflammatory factors such as Cyclooxygenase (COX)-2 and NF-kB, occurring not only in tumor progression but also in early placentation [76,77]. Curcumin appears to modulate the above-mentioned factors, influencing vessel formation by acting either as a proangiogenic or as an antiangiogenic molecule, depending on the concentration and the cell type [77]. A recent study investigated the effect of curcumin in HTR8/SVneo trophoblasts cells, a model of the human first-trimester placenta. Incubation with curcumin at low concentration (5–10 µM for 24 h) stimulated (i) proliferation with concomitant activation of Akt, (ii) tube formation of placental trophoblast HTR8/SVneo cells, (iii) and increased the expression of the proangiogenic factors VEGF, VEGFR2, and FABP4. In addition, curcumin treatment strongly increased the mRNA and protein expression of HLA-G, involved in the immune regulation during trophoblast invasion; and mRNA expression of a relevant number of genes related to the NOTCH-signaling pathway, which regulates angiogenesis. The authors examined the promoter methylation of genes involved in metabolic and oxidative stress and observed that curcumin induced hypomethylation in genes involved in the protection against oxidative stress and DNA damage. Altogether these data indicate that curcumin is able to promote angiogenesis and to activate protective pathways in the first trimester of pregnancy, and supports the development of the placental trophoblast [15]. Moreover, HTR8/SVneo trophoblast cells were used to evaluate the protective effects of curcumin against oxidative stress induced by H_2_O_2_ (400 µM for 24 h). Results showed that pretreatment with curcumin (5 µM for 24 h) increased cell viability, upregulated the activities of the antioxidant enzymes CAT and glutathione peroxidase (GSH-Px), reduced the H_2_O_2_-induced ROS accumulation and the apoptotic rate. At molecular levels, these data were associated with an increased nuclear translocation of the antioxidant transcription factor Nrf2, and reduced expression of cleaved-caspase 3 [52].

The anti-inflammatory activity of curcumin has been also observed in vitro in human gestational tissues treated with LPS. Specifically, incubation with curcumin (60 µM for 24 h) reduced IL-6 release, and IL-6 and IL-8 mRNA expression induced by LPS, in both placenta and fetal membranes. Moreover, curcumin decreased placental COX-2 mRNA expression, prostaglandin PGE2 and PGF2a release, and the expression and activity of the matrix-degrading enzyme MMP-9, in association with reduced activation of NF-kB [53].

Although several clinical trials emphasized the benefits of curcumin in different pathological contexts [10,11,32,78], there are few data on curcumin supplementation in human pregnancy. Recently, a double-blind randomized clinical trial involving 47 pregnant women with preeclampsia was conducted to evaluate the possible effect of curcumin on the expression of COX-2 and IL-10, thought to have a role in the pathogenesis of PE. The enrolled patients were randomized to receive either curcumin 100 mg/d (*n* = 23) or placebo (*n* = 24) [54]. The authors analyzed the circulating levels of IL-10 and COX-2, at T0, 90 min after curcumin ingestion, and 12 h after delivery. Results showed that curcumin did not modify the expression of the analyzed molecules at any tested time. The authors hypothesized that the absence of effect might be due to the low dose of curcumin, taking into account that in non-pregnant subjects doses can reach more than 1 g/day [54].

### 3.3. Postpartum Depression

During the antenatal and postpartum periods, women are particularly prone to develop mental disorders, including depression. Postpartum depression (PPD) occurs in 10–20% of women, leading to significant health consequences for both mother and offspring [79]. This condition has been largely underestimated and understudied so far. Hence, its prevalence is supposed to be higher, conceivably reaching 50% of women. Symptoms of depression begin during pregnancy in about 30% of women and numerous environmental, genetic, biochemical, and epigenetic factors likely contribute to the onset of PPD [79,80,81], although the exact mechanisms responsible for this condition are not yet completely known. Several pharmacological and psychological approaches are currently adopted to treat PPD, even though complementary and alternative medicine have also been taken into consideration. Increasing data have suggested the neuroprotective roles of a healthy diet, rich in fruit and vegetables, highlighting its positive influence on mental health [82]. On the contrary, an unhealthy dietary pattern increases the risk of systemic low-grade inflammation and neuroinflammation, known to be associated with PPD [18]. The neuroprotective and antidepressant benefits of curcumin have been known for a long time [83,84,85]. Several preclinical studies have suggested potential positive effects of curcumin in treating neurological disorders, such as Alzheimer’s disease, Parkinson’s disease, multiple sclerosis, migraine, epilepsy, brain and spinal cord injury, and depression [17,86,87]. Lopresti and colleagues have investigated the effects of curcumin on depression outcomes in humans. They observed that eight-week curcumin supplementation (500 mg twice a day) in subjects with major depressive disorder (MDD) was effective in reducing depressive and anxiety symptoms, as demonstrated by the reduction in total depressive symptoms (total IDS score), mood/cognitive depressive symptoms (IDSm), arousal-related symptoms (IDSa), and trait anxiety (STAIt) [88]. This supplementation resulted in an increase in urinary levels of both the arachidonic acid metabolite thromboxane B2 (Tbx-B2) and the neuropeptide substance P (SUB-P), potentially involved in depression mechanisms. Moreover, although curcumin did not modify plasma levels of endothelin-1 and leptin, a greater antidepressant benefit was observed in subjects with the highest baseline levels of these molecules. The authors hypothesized that curcumin might act by increasing endothelin and leptin receptor activities [89]. Similarly, in another trial, 1000 mg/day curcumin ingestion for six weeks or the administration of the antidepressant drug fluoxetine showed comparable efficacy in subjects with MDD [90]. A recent meta-analysis provided relevant information about curcumin use in depression. Specifically, this analysis revealed that curcumin administration (i) appears to be more effective in reducing depression symptoms at a higher dosage (1 g/day) and for six weeks or more; (ii) can enhance the action of antidepressants; and (iii) has more effects on subjects with major depression and without other comorbidities [86]. These results indicate the need for further study to better comprehend the mechanisms of action of curcumin in depression treatment.

Data obtained from animal and in vitro studies have indicated that curcumin might exert antidepressant activity by acting on different signaling pathways involved in mental disorders. Specifically, this compound is able to ameliorate the hypothalamic-pituitary-adrenal (HPA) axis disturbances [91]. Curcumin can influence the unbalanced release of monoamine neurotransmitters—such as serotonin (5-HT), dopamine (DA), noradrenaline, and glutamate—the expression of monoamine oxidase (MAO), the expression of neurotrophic factors such as brain-derived neurotrophic factor (BDNF) and neurogenesis, as well as the dysregulated immune system function and oxidative and nitrosative stress. Thus, curcumin appears to promote neurogenesis and inhibit neuronal cell apoptosis [83,84,92,93]. Despite the consistent evidence of efficacy and safety of curcumin treatment in other pathological conditions, to date, data on its effects on depression in pregnancy are completely lacking. However, in the last years, there has been a growing awareness of the possible role of anti-inflammatory micronutrients in improving PPD symptoms [18].

### 3.4. Fetal Growth and Development

According to the theory of the fetal origin of adult diseases (FOAD) hypothesized by David Barker, the intrauterine environment has a relevant role in fetal growth and development and influences disease susceptibility in the offspring in the short and long term [94]. The physiological processes of pregnancy require immune and metabolic modifications to accommodate the growing fetus; maternal malnutrition negatively influences this dynamic equilibrium, leading to tissue-specific impairment, with serious adverse outcomes for both mother and child [3,6]. Taking into consideration the importance of nutrition in human development, there is a need for better understanding the nutritional programming and the related mechanisms and players acting during pregnancy.

The placenta has the fundamental role of transferring nutrients to the fetus, and alterations in placental function have severe effects on fetal growth. Placental insufficiency is the most common cause of fetal growth restriction (FGR), a serious condition that affects 3–7% of all newborns [95]. Although the pathophysiology of FGR is not completely known, excessive oxidative stress and inflammation, as well as the activation of a complex network of several signaling pathways, appear to be involved [95,96]. The antioxidant and anti-inflammatory effects exerted by curcumin on the placenta [53] were confirmed in a mouse model of FGR fed with a low-protein (LP) diet [16]. The authors showed that maternal supplementation with curcumin (100 mg/kg day, from 1.5 to 19.5 GD) induced a potent antioxidant response in LP-fed pregnant mice; specifically, curcumin (i) increased GSH-Px activity, Nfr2 mRNA expression, and the blood sinusoids area; (ii) reduced malondialdehyde (MDA) content and apoptosis in the placenta, leading to increased placental efficiency; and (iii) elevated the expression of the antioxidant genes SOD1, SOD2, and CAT, and protein expression of Nrf2 and eme oxygenase-1(HO-1) in the liver. Overall, curcumin supplementation during pregnancy was able to revert tissue damage and contrast the decrease in fetal weight induced by a LP diet [16]. Curcumin appeared to improve birth weight, inflammation, and oxidative damage also in FGR newborn rats. Indeed, FGR rats supplemented with 400 mg/kg curcumin (at six weeks of age for six weeks) displayed reduced levels of the inflammatory cytokines TNF-α, IL-1β, and IL-6, reduced activity of AST, ALT, and MDA enzymes, and increased Gpx and GSH activity in serum. Antioxidant defense in the liver was significantly improved as well. The attenuation of the inflammatory status induced by curcumin was associated with (i) reduced activation of NF-kB and JAK2; (ii) increased expression of the antioxidant genes (Nqo1, Hmox1, Gst, Gpx1, and Sod1), and activation of their regulatory transcription factor Nfr2, in the liver [55]. Successively, the same authors investigated the effects of curcumin on insulin resistance (IR) and hepatic lipid accumulation in FGR newborn rats. Specifically, supplementation with 400 mg/kg curcumin (at six weeks of age for six weeks) attenuated IR by reducing serum insulin, glycemia, and homeostasis model assessment of insulin resistance (HOMA-IR). Furthermore, in the liver, curcumin diminished total cholesterol, TG, and non-esterified fatty acids (NEFA); increased glycogen concentration and induced the activation of lipolytic enzymes, together with a reduction in IRS-1 and Akt phosphorylation, a decrease in CD36, SREBP-1, and FASN expression, and an increase in PPARα levels. Overall, these data showed that curcumin could improve IR and lipid accumulation in the liver by regulating insulin signaling pathways, and promoting lipolysis and fatty acid oxidation in FGR rats [56].

Of note, curcumin alleviated also jejunum damage in FGR growing pigs. Indeed, the addition of 200 mg/kg curcumin to diet improved antioxidant defense (i.e., increased SOD and decreased MDA activity), immune-related gene expression (reduced mRNA of TNFα, IL-6, and IFNγ, and increased IL-2), and decreased apoptotic genes, such as caspase3 and Bax in the jejunum. Moreover, curcumin supplementation increased mRNA expression of the tight junction-related gene ocln [97].

Preterm birth (PTB) is a pregnancy complication that affects about 11% of births worldwide and is associated with increased maternal and neonate morbidity and mortality [98]. An altered inflammatory status appears to be associated with PTB. Thus the anti-inflammatory activity of curcumin has been evaluated in a mouse model of PTB, obtained through LPS injection in the abdominal cavity [57]. The injection of 100 mg/kg curcumin into the abdominal cavity, one day before (preventative group) or one day after (treatment group) LPS treatment, significantly reduced serum levels of TNF-α, IL-8, and MDA, and increased SOD levels, in both the experimental conditions, in pregnant mice. The staining intensity of NF-κB p65 showed that curcumin was able to reduce the LPS-induced expression of this inflammatory transcription factor in placental tissue both in the preventative and in the treatment group [57].

### 3.5. Toxicant Agents

Besides maternal nutrition, many other factors, including exposure to chemical and natural toxic agents, drugs, alcohol, smoking, and maternal stress influence fetal growth and development [99]. Among the myriad of properties, curcumin appears to be able to reduce toxicity induced by several environmental agents in different organs and tissues, including the brain and liver [12].

Bisphenol-A (BPA) is a chemical substance adapted to produce plastic. It has been considered an endocrine disruptor by the European Chemicals Agency (ECHA 2017) [100] due to its estrogenic activity. BPA exposure in pregnancy is associated with negative outcomes, including impaired fetal growth and childhood adiposity [101].

Remarkably, this synthetic compound affected the processes of neurogenesis in the hippocampus of the developing rat brain, and curcumin treatment showed neuroprotective activity by reverting BPA-induced effects. Specifically, pups from a pregnant rat receiving BPA (40 µg/kg body weight/day from GD6 to PND28) were treated with curcumin (200 mg/kg body weight/day from PND7 to PND28). The authors performed accurate experiments on embryo and pup brains and examined the expression of genes and pathways involved in neurogenesis. They observed that curcumin attenuated the BPA-induced reduction in neuronal stem cells (NSC) proliferation and differentiation. At molecular levels, the improvement in neurogenesis was associated with the enhanced expression of the proneural transcription factors neurogenin and neuroD1, the reduced expression of the proapoptotic molecule Bax, the increased expression of the antiapoptotic molecule Bcl-2, and the activation of Wnt/βcatenin signaling that regulates NSC proliferation and differentiation. Of note, the benefits of curcumin resulted in improved learning and memory in BPA-treated pups [58].

Mercury (Hg) is a widely diffused toxic heavy metal that occurs naturally in three forms, namely metallic Hg, organic Hg, and inorganic Hg. Human exposure to Hg occurs mainly through the environment (e.g., mercury-contaminated sea fish, dental amalgam). Of note, occupation (e.g., mining) is another important source of exposure for humans and is associated with possible multi-organ toxicity [102]. As for the influence of Hg on neurodevelopment, a cross-sectional study, involving healthy Saudi mothers and their infants (age 3–12 months), showed an association between Hg exposure and neurodevelopmental delay, with possible negative effects persisting also in adulthood [103]. Interestingly, curcumin appeared to mitigate Hg toxicity in animal models [102]. Specifically, pregnant mice were exposed (from 1GD to 15PND) to 10 ppm mercuric chloride (HgCl_2_) in the presence or absence of 150 and 300 ppm curcumin. Hg exposure induced serious damage to the development of neuromotors, and increased anxiety behavior in pups. Curcumin administration improved neurodevelopment and reduced anxiety, by restoring the levels of neurotransmitters DA, 5-HT, and acetylcholinesterase (AChE), and of the antioxidant GSH, decreased by Hg exposure, in forebrain pups [59]. Moreover, by using the same experimental conditions, the authors analyzed changes in body weight, sexual behavior, and fertility in male and female pups. The obtained data showed that curcumin counteracted the perinatal effects of Hg exposure by increasing (i) body weight, liver and brain weight in male and female pups; (ii) epididymis, seminal vesicle, testis weight in males; and (iii) ovary weight in females; also sexual behavior was improved in both sexes. Moreover, curcumin increased testosterone and FSH levels, and sperm motility in males, as well as FSH, LH, and progesterone in females, reduced by Hg exposure [60].

Lead (Pb) is a heavy metal widely spread in the environment. It is extremely dangerous for both animals and humans. Lead exposure occurs mainly through food and water contamination, and air pollution. Lead can cross the placental and blood-brain barrier, inducing neurotoxicity. Curcumin exerted neuroprotective effects contrasting lead-induced damage in rats. The concomitant exposure of rat mothers to Pb (3 g/L) and curcumin (16 g/kg) during pregnancy and lactation resulted in the recovery of the Pb-induced altered sensorimotor functions in neonatal rats. Pb neurotoxicity produced alterations in locomotor neuronal network development and curcumin treatment reversed these anomalies, allowing normal locomotor behavior. These findings indicate that curcumin has the capability to prevent central nervous system dysfunction induced by lead during the earlier stages of development [61].

Celecoxib is a selective inhibitor of COX-2 that is able to reduce pain and inflammation caused by several inflammatory conditions [62]. Since recent data have shown that the inhibition of COX-2 reduced adult neural cell proliferation and differentiation [104], Wang et al., investigated the neuroprotective action of curcumin on fetal brain development in pregnant mice treated with celecoxib [62]. Specifically, pregnant mice were pretreated with curcumin (500 nmol/kg body weight) from embryonic day (E) 13.5 to E16.5, and then with celecoxib (300 mg/kg body weight) from E16.5 to E17.5. Results showed that curcumin counteracted the celecoxib-induced inhibition of neurogenesis in the fetal frontal cortex, by increasing proliferation and Cyclin D1 expression in neural progenitor cells, and by activating Wnt/βcatenin signaling (i.e., decreased expression of glycogen synthase kinase 3 beta (GSK-3β), and increased expression of βcatenin) [62].

Valproic acid (VPA), a branched short-chain fatty acid, is an antiepileptic agent that has been associated with congenital malformations, including alterations in fetal brain development, and consequent intellectual disabilities and autistic spectrum disorders in the offspring [105]. Curcumin appears to attenuate the VPA-induced brain damage, as observed in a rodent model of autism. Neonatal rats, born to mothers treated with VPA from 12.5 gestational day, received a single dose of curcumin (1 g/kg day), and their brains were analyzed 28 days after birth. Curcumin was able to ameliorate body and brain weight, and the altered expression of IL-6, IFN-γ, GSH, CYP450, in the brain of VPA-exposed pups [63].

Prenatal alcohol exposure (PAE) has dramatic effects on fetal growth and development (fetal alcohol spectrum disorders: FASD) and is responsible for neurodevelopmental disorders (i.e., neurocognitive and behavioral deficits, and increased susceptibility to mental health disorders) and birth defects (growth deficits and physical abnormalities). PAE induces chromosomal rearrangements and epigenetic alterations, therefore leading to altered gene-environment interactions that are responsible for alcohol-induced disorders [106]. Curcumin (100 mg/kg body weight), administered during the peri-adolescence period (PND 28–35), appeared to counteract fetal brain damage induced by prenatal and lactational alcohol exposure (PLAE; 20% (*v*/*v*) alcohol solution) in mice. The authors showed that curcumin improved anxiety and memory deficits caused by PLAE, and these improvements were associated with reduced microglia activation and astrogliosis. At molecular levels, curcumin reduced protein expression of IL-6, TNF-α, and NF-kB. These data showed that curcumin may act against cognitive deficits and neuroinflammation induced by alcohol exposure in pregnancy [64].

Curcumin can counteract the deleterious effects of PAE on cardiac development, as demonstrated in a mouse model. Pregnant mice were daily exposed to ethanol (56% *v*/*v* in saline) between embryonic days 7.5 to 15.5; at embryonic day 17.5, mice were euthanized and embryonic hearts were removed. Results showed that PAE treatment increased apoptosis in pup hearts; this finding was associated with higher levels of caspase-3 and -8 mRNA expression, and reduced Bcl-2 mRNA expression, due to a different modulation of histone H3K9 acetylation near the promoter regions of caspase-3, caspase-8 (hyperacetylation), and Bcl-2 (hypoacetylation). In vitro, curcumin (25 µM for 24 h) treatment abolished apoptosis and reverted the expression of caspases and Bcl-2, induced by alcohol (200 mM), in cardiac progenitor cells. These results highlighted the capability of curcumin to prevent congenital heart diseases induced by PAE in pregnancy, by acting as an epigenetic modulator [65].

### 3.6. Adverse Effects on Embryos

Embryonic development is a complex process that is finely regulated and highly susceptible to environmental influences. Therefore, it is reasonable to hypothesize that the anti-inflammatory, antioxidative, antiproliferative, and antiangiogenic properties of curcumin could interfere with the blastocyst stage, implantation and post-implantation development of embryos [66].

Chen and colleagues evaluated the possible embryotoxicity of curcumin in mouse blastocysts both in vitro and in vivo. They observed that curcumin (24 μM for 24 h) induced apoptosis in mouse blastocysts, and reduced implantation rate and development, in vitro. Then, embryos treated with curcumin were transferred in vivo; results confirmed a significant reduction in implantation ratio, and, among the implanted embryos, a higher rate of failure to develop normally. The authors evaluated the possible mechanisms responsible for these effects and found that curcumin-induced apoptosis was associated with the modulation of pro- and anti-apoptotic molecules (i.e., increased Bax and reduced Bcl-2 expression), ROS generation, and caspase-3 activation [66]. Additionally, the same authors showed that curcumin (24 μM) adversely affected oocytes maturation, in vitro. This effect resulted in a reduced ability of oocytes to be fertilized, increased blastocyst apoptosis, and reduced blastocyst implantation ratio and development. These results were confirmed in oocytes collected from female mice after feeding them with curcumin supplementation (40 μM) for four days [67]. Another in vitro study highlighted that the degree of damage induced by curcumin (6, 12, or 24 μM curcumin for 24 h) on mouse blastocyst at the implantation stage and during the early post-implantation stage is dose-dependent. Specifically, 6 μM and 12 μM curcumin inhibited cell proliferation of the blastocyst but increased the formation of trophoblastic giant cells, whereas 24 μM curcumin exposure was lethal to all blastocysts, and induced severe damage to the implanted blastocysts [68].

Further evidence on these effects comes from a recent study in zebrafish. The exposure of zebrafish embryos and larvae to different concentrations of Curcuma Longa extract (7.80, 15.63, 31.25, 62.50, 125.0, and 250.0 μg/mL) at different hours post fertilization (hpf: 24, 48, 72, 96, 120 h) showed that a dosage above 62.50 μg/mL had toxic effects, and a dosage of 125.0 μg/mL increased embryo mortality and induced morphological deformities in larvae [19]. Despite the potential benefits of curcumin described in different pathological conditions, all these data indicate that dosage and time of exposure throughout pregnancy should be carefully evaluated to avoid serious damage to embryo development.

## 4. Conclusions and Future Perspectives

The use of the natural product curcumin to treat medical conditions is spreading around the world. There is an increasing public interest in the potential health benefits of this compound, as evidenced by the large number of currently available curcumin formulations, aimed at increasing its bioavailability and efficacy, and by the considerable number of scientific papers published over the last years.

This review has drawn attention towards the effects of curcumin on pregnancy and pregnancy complications, considering that during gestation, mother and fetus undergo significant (patho-) physiological changes.

Almost all data emphasizing the numerous biological activities of curcumin have been obtained from pregnant rodents and in vitro studies. Curcumin appeared to ameliorate diabetes in a GDM mouse model, as well as PE in a PE rat model, and was found to be neuroprotective against environmental toxic agents. The antidepressant activity of curcumin has also been tested in humans. However, to date, studies on the possible beneficial effects of curcumin on PPD, a largely underestimated and understudied condition, are completely lacking. As regards fetal growth and development, curcumin counteracted the modifications associated with FGR and PTB in rodent models but negatively affected blastocyst stage, implantation and post-implantation embryo development in healthy animals.

Altogether, these results indicate that the use of curcumin in pregnancy must be carefully evaluated. The growing use of curcumin as self-medication along with the misleading perception that “natural” is the equivalent of “safe” are additional issues of concern.

Further studies are needed to clarify whether pregnancy might benefit from curcumin’s properties; for this purpose, the collaboration between multidisciplinary scientific teams is essential to provide a holistic view of the complex networks between natural products and human physiology. Systems biology and the recently developed network pharmacology represent new strategies to better comprehend the mechanisms underlying curcumin activities in the human body.

## Figures and Tables

**Table 1:** Effects of curcumin on pregnancy and pregnancy-related disorders.

Curcumin	Experimental Model	Outcomes	References
**Altered glucose metabolism**			
100 mg/kg/day (from 0 to 20 GD)	Mouse model of GDM	↓Maternal glucose and insulin levels; improved oxidative stress (↑ GSH, SOD, CAT), and ↑AMPK and ↓HDAC4, in the liver; restored offspring litter size and body weight	Lu, X., 2019 [14]
20 μM for 24 h	Mouse embryos (E8.5 of development) cultured for 24 h with 100 mg/dL glucose	↓Neural tube defects by reducing oxidative stress (↓4-HNE, ↓LPO, ER stress (↓p-PERK, p-IRE1α, p-eIF2α, CHOP, BiP and XBP1 expression), and apoptosis (↓caspase-3 and -8 cleavage)	Wu, Y., 2015 [49]
**Cardiovascular disorders**			
0.36 mg/kg/day (from 0 to GD18)	Rat model of PE (LPS-induced)	Improved hypertension, proteinuria, and renal damage; ↓serum levels of IL-6 and MCP-1; ↓ placental TLR4, IL-6, and NFkB expression; improved trophoblast invasion and spiral artery remodeling	Gong, P., 2016 [50]
0.36 mg/kg/day (from 0.5 to GD18)	Mouse model of PE (LPS-induced)	↑Number of live pups, and fetal and placental weight; ↓inflammation (↓TNF-α, IL-1β, IL-6, MCP-1, and MIP-1 placental expression), ↑ Akt activation	Zhou, J., 2017 [51]
5–10 µM for 24 h	HTR8/SVneotrophoblast cells (model for human first-trimester placenta)	↑Proliferation associated with Akt activation, ↑tube formation; ↑proangiogenic factors VEGF, VEGFR2, and FABP4 expression; ↑ expression of NOTCH-signaling pathway mediators; ↑promoter hypomethylation of oxidative and metabolic stress genes	Basak, H., 2020 [15]
5 µM for 24 h	HTR8/SVneo trophoblast cells (H_2_O_2_-treated)	↑Cells viability; ↓oxidative stress (↑CAT, GSH-Px activities); ↑Nrf2 activation and ↓ caspase-3 activation	Qi, L., 2020 [52]
60 µM for 24 h	Human placental and fetal membranes, LPS-treated	↓IL-6, IL-8, and COX-2 mRNA expression; ↓PGE2 and PGF2a release; ↓MMP-9 expression and NFkB activation	Lim, R., 2013 [53]
100 mg (single dose)	47 pregnant women with PE	No differences in serum level of COX-2 and IL-10	Fadinie, W., 2019 [54]
**Fetal growth and development**			
100 mg/kg/day (from 1.5 to 19.5 GD)	Mouse model of FGR (low-protein diet)	↓Placental apoptosis and ↑ placental blood sinusoids area; ↑GSH-Px activity, Nfr2 mRNA expression; ↑antioxidant genes expression (SOD1, SOD2, CAT, Nrf2, and HO-1), in fetal liver	Qi, L., 2020 [16]
400 mg/kg/day at 6 weeks of age for 6 weeks	FGR newborn rats	↓TNF-α, IL-1β and IL-6 levels, ↓activity of AST, ALT, and MDA, ↑Gpx and GSH activity, in serum;↓NF-kB and JAK2 expression, ↑antioxidant genes (Nqo1, Hmox1, Gst, Gpx1 and Sod1), an Nfr2 activation, in the liver	He, J., 2018 [55]
400 mg/kg/day at 6 weeks of age for 6 weeks	FGR newborn rats	↓Glucose levels and IR; ↓TAG, NEFA, total cholesterol, ↑glycogen (↓IRS-1 and Akt phosphorylation, CD36, SREBP-1, and FASN expression, ↑PPARα), in the liver	Niu, Y., 2019 [56]
100 mg/kg (single dose)	Mouse model of PTB, LPS-induced	↓TNF-α, IL-8, MDA, and ↑SOD serum levels; ↓NFkB activation in placenta	Guo, Y.Z., 2017 [57]
**Toxicant agents**			
200 mg/kg/day (from 7 to PND28)	Pregnant rats, BPA-treated	Neuroprotective; ↑proliferation and differentiation of neuronal stem cells (↑neurogenin and neuroD1 expression); ↓apoptosis (↓Bax, ↑Bcl-2 expression); improvement in learning and memory	Tiwari, S.K., 2019 [58]
150/300 ppm/day (from GD1 to 15PND)	Pregnant mice, HgCl_2_-treated	↑Neurodevelopment and ↓anxiety (↑levels of DA, 5-HT, AChE, and GSH)	Abu-Taweel, G.M., 2019 [59]
150/300 ppm/day (from GD1 to 15PND)	Pregnant mice, HgCl_2_-treated	↑Pups body weight; ↑male genitalia weight, testosterone, and FSH levels; ↑ovary weight and progesterone, FSH and LH levels; improved sexual behavior in both sexes	Abu-Taweel, G.M., 2020 [60]
16 g/kg/day during pregnancy and lactation	Pregnant rats, Pb-treated	Prevented central nervous system dysfunction allowing normal locomotor behavior	Benammi, H., 2017 [61]
Pretreatment with curcumin 500 nmol/kg/day (from ED 13.5 to E16.5)	Pregnant mice, celecoxib-treated	↑Neurogenesis in fetal frontal cortex (↑Cyclin D1 expression, and activation of Wnt/βcatenin signaling in neural progenitor cells)	Wang, R., 2017 [62]
Single-dose curcumin (1 g/kg) in neonatal rats	Pregnant rats, VPA-treated	↑Body and brain weight in pups; ↓IL-6, IFN-γ, and ↑GSH, CYP450 expression, in brain pups	Al-Askar, M., 2017 [63]
Offsprings 100 mg/kg/day (from 28 to 35 PND	PLAE-pregnant mice (offspring peri-adolescence period)	Improved offspring anxiety and memory deficits; ↓Neuroinflammation (↓IL-6, TNF-α, and NF-kB expression)	Cantacorps, L., 2020 [64]
Embryos 25 µM for 24 h	PAE-pregnant mice (embryos E17.5)	Improved offspring anxiety and memory deficits; ↓neuroinflammation (↓IL-6, TNF-α, and NF-kB expression)	Yan, X., 2017 [65]
**Adverse effects on embryos**			
24 μM for 24 h	Mouse blastocysts	↑Apoptosis (↑Bax and ↓Bcl-2 expression); ↓ implantation rate and development	Chen, C.C., 2010 [66]
24 μM for 24 h	Mouse oocytes	↑Apoptosis; ↓ oocytes fertilization; ↓implantation rate and development	Chen, C.C., 2012 [67]
6–24 μM for 24 h	Mouse blastocysts (at implantation stage and during the early post-implantation stage)	Dose-dependent damage, 24 μM lethal for all blastocysts	Huang, F.J., 2013 [68]
Curcuma longa extract (7.80–125 µg/mL) for 5 days	Zebrafish embryos and larvae at different hours of post-fertilization (24–120 h)	Dose-dependent toxic effects: malformations above 62.50 µg /mL, and mortality at 125.0 µg/mL	Alafiatayo, A.A., 2019 [19]

Abbreviations: ↑ Increases; ↓ Decreases; GDM, gestational diabetes mellitus; GD, gestational day; GSH, glutathione; SOD, superoxide dismutase; CAT, catalase; AMPK, 5′ AMP-activated protein chinasi; HDAC4, histone deacetylase 4; 4-HNE, 4-hydroxynonenal; LPO, lipid peroxidation; ER, endoplasmic reticulum; p-PERK, phospho-protein kinase-like endoplasmic reticulum kinase; p-IRE1α, phospho-inositol-requiring kinase 1α; p-eIF2α, phospho-eukaryotic Initiation Factor 2α; CHOP, C/EBP homologous protein; BiP, binding immunoglobulin protein; XBP1, X-box-binding protein-1; PE, preeclampsia; LPS, lipopolysaccharides; IL6, interleukin-6; MCP-1, monocyte chemoattractant protein-1; TLR4, toll-like Receptor 4; NFkB, nuclear transcriptor factor kappa B; TNFα, tumor necrosis factor α; IL1β, interleukin-1β; MIP-1, macrophage inflammatory protein-1; Akt, protein kinase B; VEGF, vascular endothelial growth; VEGFR2, vascular endothelial growth factor receptor 2; FABP4, fatty acid binding protein 4; GSH-Px, glutathione peroxidase; Nrf2, nuclear factor erythroid-2-related factor-2; IL-8, interleukin-8; COX-2, cyclooxigenase-2; PGE2, prostaglandin E2; PGF2a, prostaglandin F2α; MMP-9, metalloproteinase-9; IL-10, interleukin-10; FGR, fetal growth restriction; HO-1, heme oxygenase-1(enzyme); AST, aspartate aminotransferase; ALT, aminotransferase; MDA, malondialdehyde; JAK2, Janus kinase 2; Nqo1, quinone dehydrogenase; Hmox1, heme oxygenase 1 (gene); Gst, glutathione S-transferase; Gpx1, glutathione peroxidase; IR, insulin resistance; TAG, triglycerides; NEFA, Non-Esterified Fatty Acids; IRS-1, insulin receptor substrate-1; PTB, preterm birth; CD36, cluster of differentiation 36; SREBP-1, stearoyl CoA desaturase-1; FASN, Fatty acid synthase; PPARα, Peroxisome Proliferator Activated Receptors-α; PND, postnatal day; BPA, bisphenol-A; DA, dopamine; 5-HT, serotonin; AChE, acetylcholinesterase; FSH, follicle stimulating hormone; LH, luteinizing hormone; ED, embrionic day; Pb, plumbum (lead); VPA, valproic acid; IFN-γ, interferon γ; CYP450, cytochromes P450; PLAE, prenatal and lactational alcohol exposure; PAE, prenatal alcohol exposure; PND, postnatal day; B-cell lymphoma protein 2 (Bcl-2)-associated X (Bax); B-cell lymphoma protein 2 (Bcl-2).
